# Biomarkers for the early detection of acute kidney injury

**DOI:** 10.1007/s00467-007-0470-x

**Published:** 2008-12-01

**Authors:** Mai T. Nguyen, Prasad Devarajan

**Affiliations:** Nephrology and Hypertension, MLC 7022, Cincinnati Children’s Hospital Medical Center, 3333 Burnet Avenue, Cincinnati, OH 45229-3039 USA

**Keywords:** Acute renal failure, Lipocalin, Cystatin C, Interleukin 18, Kidney injury molecule-1, Biomarker

## Abstract

Acute kidney injury (AKI), previously referred to as acute renal failure (ARF), represents a persistent problem in clinical medicine. Despite significant improvements in therapeutics, the mortality and morbidity associated with AKI remain high. A major reason for this is the lack of early markers for AKI, akin to troponins in acute myocardial disease, and hence an unacceptable delay in initiating therapy. Fortunately, the application of innovative technologies such as functional genomics and proteomics to human and animal models of AKI has uncovered several novel genes and gene products that are emerging as biomarkers. The most promising of these are chronicled in this article. These include a plasma panel [neutrophil gelatinase-associated lipocalin (NGAL) and cystatin C] and a urine panel [NGAL, interleukin 18 (IL-18), and kidney injury molecule 1 (KIM)-1]. As they represent sequentially expressed biomarkers, it is likely that the AKI panels will be useful for timing the initial insult and assessing the duration of AKI. Based on the differential expression of the biomarkers, it is also likely that the AKI panels will distinguish between the various types and etiologies of AKI. It will be important in future studies to validate the sensitivity and specificity of these biomarker panels in clinical samples from large cohorts and from multiple clinical situations.

## Introduction

Acute kidney injury (AKI), previously referred to as acute renal failure (ARF), represents a significant and devastating problem in clinical medicine [1–3]. The incidence of AKI varies from 5% of hospitalized patients to 30–50% of patients in intensive care units, and there is now substantial evidence that the incidence is rising at an alarming rate [4]. Despite significant improvements in therapeutics, the mortality and morbidity associated with AKI remain high. Outstanding advances in basic research have illuminated the pathogenesis of AKI and have paved the way for successful therapeutic approaches in animal models [2]. However, translational research efforts in humans have yielded disappointing results. A major reason for this is the lack of early markers for AKI, akin to troponins in acute myocardial disease, and hence an unacceptable delay in initiating therapy [5, 6]. In current clinical practice, AKI is typically diagnosed by measuring serum creatinine. Unfortunately, creatinine is an unreliable indicator during acute changes in kidney function [7]. First, serum creatinine levels can vary widely with age, gender, lean muscle mass, muscle metabolism, and hydration status. Second, serum creatinine concentrations may not change until about 50% of kidney function has already been lost. Third, at lower rates of glomerular filtration, the amount of tubular secretion of creatinine results in overestimation of renal function. Finally, during acute changes in glomerular filtration, serum creatinine does not accurately depict kidney function until steady-state equilibrium has been reached, which may require several days. However, animal studies have shown that whereas AKI can be prevented and/or treated by several maneuvers, these must be instituted very early after the insult, well before the rise in serum creatinine [2, 5]. The lack of early biomarkers of AKI in humans has negatively impacted on a number of landmark clinical trials investigating highly promising therapies for AKI in adults [8, 9]. In addition, pediatric continuous renal replacement therapy (CRRT) studies have shown an association between increasing degrees of fluid overload at the time of CRRT initiation and mortality [10]. Early predictive AKI biomarkers could identify patients who may benefit from early initiation of CRRT.

## Ideal properties and roles of biomarkers in AKI

In addition to aiding in early diagnosis and prediction, biomarkers may serve several additional purposes in AKI. Thus, biomarkers are also needed for (a) discerning AKI subtypes (prerenal, intrinsic renal, or postrenal), (b) identifying AKI etiologies (ischemia, toxins, sepsis, or a combination), (c) differentiating AKI from other forms of acute kidney disease (urinary tract infection, glomerulonephritis, interstitial nephritis), (d) predicting the AKI severity (risk stratification for prognostication as well as to guide therapy), (e) monitoring the course of AKI, and (f) monitoring the response to AKI interventions. Furthermore, AKI biomarkers may play a critical role in expediting the drug development process. The Critical Path Initiative issued by the U.S. Food and Drug Administration (FDA) in 2004 stated that “Additional biomarkers (quantitative measures of biologic effects that provide informative links between mechanism of action and clinical effectiveness) and additional surrogate markers (quantitative measures that can predict effectiveness) are needed to guide product development”. Identification of novel AKI biomarkers has been designated as a top priority by the American Society of Nephrology [11]. The concept of developing a new toolbox for earlier diagnosis of disease states is also prominently featured in the National Institutes of Health (NIH) Roadmap for biomedical research [12].

Desirable characteristics of clinically applicable AKI biomarkers include: (a) they should be noninvasive and easy to perform at the bedside or in a standard clinical laboratory using easily accessible samples such as blood or urine, (b) they should be rapidly and reliably measurable using a standardized assay platform, (c) they should be highly sensitive to facilitate early detection and with a wide dynamic range and cutoff values that allow for risk stratification, (d) they should be highly specific for AKI and enable the identification of AKI subtypes and etiologies, and (e) they should exhibit strong biomarker properties on receiver-operating characteristic (ROC) curves.

**Fig. 1:**
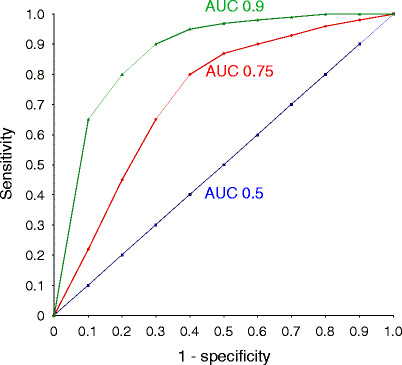
Three hypothetical receiver-operating characteristic (ROC) curves are shown. The* blue* (*straight*) *line* represents a biomarker with an area under the curve (AUC) of 0.5, which indicates a result that is no better than expected by random chance. The* red* (*middle*) *curve* yields an AUC of about 0.75, which is generally considered a good biomarker. The* green* (*top*) *curve* gives an AUC of approximately 0.9, which would represent an excellent biomarker

The ROC analysis has been extensively used as a fundamental evaluation tool in clinical studies pertaining to diagnostic testing [13, 14]. An ROC curve is a graphical plot of the sensitivity on the y-axis versus (1-specificity) on the x-axis for a binary classifier system, as its discrimination threshold is varied. ROC curves can also be generated by plotting the fraction of true positives on the y-axis and the fraction of false positives on the x-axis. For biomarker analysis, the binary classification task is typically to determine whether a subject has a certain disease (such as AKI) or not. Characteristically, ROC curves are generated for various cutoff points for the biomarker concentration under consideration. Examples of hypothetical ROC curves are shown in Fig. 1. The best possible biomarker of a disease process would yield a plot that was a point in the upper left corner of the ROC space, which would represent 100% sensitivity (all true positives detected) and 100% specificity (no false positives found). A completely random biomarker would result in a straight line at a 45° angle from bottom left to top right (the line of no discrimination). A commonly derived statistic from the ROC curve is the area under the curve (AUC). An AUC of 1.0 represents a perfect biomarker, whereas an AUC of 0.5 (as would be derived from the line of no discrimination) indicates a result that is no better than expected by random chance. An AUC of 0.75 or above is generally considered a good biomarker, and an AUC of 0.9 or above would represent an excellent biomarker.

## The search for novel AKI biomarkers

The quest for AKI biomarkers is an area of intense contemporary research [15–18]. Conventional urinary biomarkers such as casts and fractional excretion of sodium have been insensitive and nonspecific for the early recognition of AKI [17]. Other traditional urinary biomarkers, such as filtered high molecular weight proteins and tubular proteins or enzymes, have also suffered from lack of specificity and dearth of standardized assays [16]. Fortunately, the application of innovative technologies such as functional genomics and proteomics to human and animal models of AKI has uncovered several novel genes and gene products that are emerging as biomarkers [15, 18]. The most promising of these are outlined in Table 1, and their current status in human AKI is chronicled in this article.

**Table 1:** Current status of promising acute kidney injury (AKI) biomarkers in various clinical situations

Biomarker Name	Sample Source	Cardiac Surgery	Contrast Nephropathy	Sepsis or ICU	Kidney Transplant	Commercial Test?
NGAL	Plasma	Early	Early	Early	Early	Biosite^a^
Cystatin C	Plasma	Intermediate	Intermediate	Intermediate	Intermediate	Dade-Behring
NGAL	Urine	Early	Early	Early	Early	Abbott^a^
IL-18	Urine	Intermediate	Absent	Intermediate	Intermediate	None
KIM-1	Urine	Intermediate	Not tested	Not tested	Not Tested	None

*NGAL* neutrophil gelatinase-associated lipocalin,* IL-18* interleukin 18,* KIM-1* kidney injury molecule 1

^a^In development

Genomics, the study of the genetic makeup of a species, gained popularity in the 1990s with the initiation of the Human Genome Project, which was completed in 2001. The human genome (which contains an estimated 25,000 genes) as well as genomes of other species has been delineated and placed into databases, which can easily be searched. The advent of the microarray, or DNA chip, allows investigators to search through thousands of genes simultaneously, making the process much more efficient. Such gene expression profiling studies have identified at least two genes whose protein products have emerged as AKI biomarkers, as detailed below [15]. However, one disadvantage of microarray-based methods is that they cannot be used for the direct analysis of biological fluids.

Proteomics is the study of both the structure and function of proteins. It is estimated that the human proteome contains over 400,000 proteins. Proteins can be studied using a variety of methods, such as gel electrophoresis, immunoblotting, mass spectrometry, and enzymatic or metabolic assays. Each method is used to determine different types of information and has its own set of strengths and limitations. Advancing technologies have radically improved the speed and precision of identifying and measuring proteins in biological fluids, and proteomic approaches are beginning to yield novel urinary biomarkers in human models of AKI [18–20].

## Novel AKI biomarkers under evaluation in humans

### Neutrophil gelatinase-associated lipocalin (NGAL)

Human neutrophil gelatinase-associated lipocalin (NGAL) was originally identified as a 25 kDa protein covalently bound to gelatinase from neutrophils. NGAL is normally expressed at very low levels in several human tissues, including kidney, lungs, stomach, and colon. NGAL expression is markedly induced in injured epithelia. For example, NGAL concentrations are elevated in the serum of patients with acute bacterial infections, the sputum of subjects with asthma or chronic obstructive pulmonary disease, and the bronchial fluid from the emphysematous lung [21]. NGAL was recently identified by microarray analysis as one of the earliest and most robustly induced genes and proteins in the kidney after ischemic or nephrotoxic injury in animal models, and NGAL protein was easily detected in the blood and urine soon after AKI [22–26]. These findings have spawned a number of translational studies to evaluate NGAL as a novel biomarker of human AKI.

In a cross-sectional study, human adults in the intensive care unit with established ARF (defined as a doubling of the serum creatinine in less than 5 days) secondary to sepsis, ischemia, or nephrotoxins displayed a greater than ten-fold increase in plasma NGAL and a greater than 100-fold increase in urine NGAL by Western blotting when compared with normal controls [25]. Both plasma and urine NGAL correlated highly with serum creatinine levels. Kidney biopsies in these patients showed intense accumulation of immunoreactive NGAL in 50% of the cortical tubules. These results identified NGAL as a widespread and sensitive response to established AKI in humans.

In a prospective study of children undergoing cardiopulmonary bypass, AKI (defined as a 50% increase in serum creatinine) occurred in 28% of subjects, but the diagnosis using serum creatinine was only possible 1–3 days after surgery [27]. In marked contrast, NGAL measurements by Western blotting and by enzyme-linked immunosorbent assay (ELISA) revealed a robust ten-fold or greater increase in the urine and plasma within 2–6 h of surgery in patients who subsequently developed AKI. Both urine and plasma NGAL were powerful independent predictors of AKI, with an outstanding AUC of 0.998 for the 2-h urine NGAL and 0.91 for the 2-h plasma NGAL measurements [27]. Thus, plasma and urine NGAL emerged as sensitive, specific, and highly predictive early biomarkers of AKI after cardiac surgery in children. It should be emphasized that the patients in this study were primarily children with congenital heart disease who lacked many of the common comorbid conditions (such as diabetes, hypertension, and atherosclerosis) that are frequently encountered in adults. Nevertheless, these findings have now been confirmed in a prospective study of adults who develop AKI after cardiac surgery, in whom urinary NGAL was significantly elevated by 1–3 h after the operation [28]. AKI, defined as a 50% increase in serum creatinine, did not occur until the third postoperative day. However, patients who did not encounter AKI also displayed a significant increase in urine NGAL in the early postoperative period although to a much lesser degree than in those who subsequently developed AKI. The AUC reported in the adult study was 0.74 for the 3-h NGAL and 0.80 for the 18-h NGAL, which is perhaps reflective of the confounding variables that typically accumulate with age.

NGAL has also been evaluated as a biomarker of AKI in kidney transplantation. Biopsies of kidneys obtained 1 h after vascular anastomosis revealed a significant correlation between NGAL staining intensity and the subsequent development of delayed graft function [29]. In a prospective multicenter study of children and adults, urine NGAL levels in samples collected on the day of transplant clearly identified cadaveric kidney recipients who subsequently developed delayed graft function and dialysis requirement (which typically occurred 2–4 days later). The ROC curve for prediction of delayed graft function based on urine NGAL at day 0 showed an AUC of 0.9, indicative of an excellent predictive biomarker [30]. Urine NGAL has also been shown to predict the severity of AKI and dialysis requirement in a multicenter study of children with diarrhea-associated hemolytic uremic syndrome [31]. Preliminary results also suggest that plasma and urine NGAL measurements represent predictive biomarkers of AKI following contrast administration [32–34] and in the pediatric intensive care setting [35].

In summary, NGAL is emerging as a center-stage player in the AKI field as a novel predictive biomarker. However, it is acknowledged that the studies published thus far are small, in which NGAL appears to be most sensitive and specific in relatively uncomplicated patient populations with AKI. NGAL measurements may be influenced by a number of coexisting variables, such as preexisting renal disease [36] and systemic or urinary tract infections [19, 21]. Large multicenter studies to further define the predictive role of plasma and urine NGAL as a member of the putative AKI panel have been initiated, robust assays for commercialization are nearly complete, and the results are awaited with optimism.

### Cystatin C

Cystatin C is a cysteine protease inhibitor that is synthesized and released into the blood at a relatively constant rate by all nucleated cells. It is freely filtered by the glomerulus, completely reabsorbed by the proximal tubule, and not secreted. As blood levels of cystatin C are not significantly affected by age, gender, race, or muscle mass, it is a better predictor of glomerular function than is serum creatinine in patients with chronic kidney disease [37]. Urinary excretion of cystatin C has been shown to predict the requirement for renal replacement therapy in patients with established AKI about 1 day earlier, with an AUC of 0.75 [38]. In the intensive care setting, a 50% increase in serum cystatin C predicted AKI 1 to 2 days before the rise in serum creatinine, with an AUC of 0.97 and 0.82, respectively [6].

A recent prospective study compared the ability of serum cystatin C and NGAL in the prediction of AKI following cardiac surgery in children [39]. Out of 129 patients, 41 developed AKI (defined as a 50% increase in serum creatinine) 1–3 days after cardiopulmonary bypass. In AKI cases, serum NGAL levels were elevated at 2 h postsurgery, whereas serum cystatin C levels increased only after 12 h. Both NGAL and cystatin C levels at 12 h were strong independent predictors of AKI, but NGAL outperformed cystatin C at earlier time points.

Thus, both NGAL and cystatin C represent promising sequential biomarker candidates for inclusion in the blood AKI panel. An advantage of cystatin C is the commercial availability of a standardized immunonephelometric assay, which is automated and provides results in minutes. Additionally, routine clinical storage conditions, freeze/thaw cycles, the presence of interfering substances, and the etiology of the AKI do not affect serum cystatin C measurements.

### Kidney injury molecule 1 (KIM-1)

Kidney injury molecule 1 (KIM-1) is a transmembrane protein that is highly overexpressed in dedifferentiated proximal tubule cells after ischemic or nephrotoxic AKI in animal models [40, 41], and a proteolytically processed domain is easily detected in the urine [42]. In a small human cross-sectional study, KIM-1 was found to be markedly induced in proximal tubules in kidney biopsies from patients with established AKI (primarily ischemic), and urinary KIM-1 distinguished ischemic AKI from prerenal azotemia and chronic renal disease [40]. Patients with AKI induced by contrast did not have increased urinary KIM-1.

Recent preliminary studies have expanded the potential clinical utility of KIM-1 as a predictive AKI biomarker. In a cohort of 103 adults undergoing cardiopulmonary bypass, AKI (defined as a 0.3 mg/dl increase in serum creatinine) developed in 31%, in whom the urinary KIM-1 levels increased by about 40% at 2 h postsurgery and by more than 100% at the 24-h time point [43]. In a small case-control study of 40 children undergoing cardiac surgery, 20 with AKI (defined as a 50% increase in serum creatinine) and 20 without AKI, urinary KIM-1 levels were markedly enhanced, with an AUC of 0.83 at the 12-h time point [44].

Thus, KIM-1 represents a promising candidate for inclusion in the urinary AKI panel. An advantage of KIM-1 over NGAL is that it appears to be more specific to ischemic or nephrotoxic kidney injury and is not significantly affected by chronic kidney disease or urinary tract infections. It is likely that NGAL and KIM-1 will emerge as temporally sequential biomarkers of AKI, with NGAL being most sensitive at the earliest time points and KIM-1 adding significant specificity at slightly later time points.

### Interleukin 18 (IL-18)

Interleukin 18 (IL-18) is a proinflammatory cytokine that is induced and cleaved in the proximal tubule and subsequently easily detected in the urine following ischemic AKI in animal models [45]. In a cross-sectional study, urine IL-18 levels were markedly increased in patients with established AKI but not in subjects with urinary tract infection, chronic kidney disease, nephritic syndrome, or prerenal failure [46]. Urinary IL-18 levels displayed sensitivity and specificity of >90% for the diagnosis of established AKI. In addition, IL-18 in urine obtained on the day of kidney transplantation was significantly increased in patients who subsequently developed delayed graft function, with an AUC of 0.90 [30]. Urinary IL-18 was significantly upregulated up to 48 h prior to the increase in serum creatinine in patients with acute respiratory distress syndrome who develop AKI, with an AUC of 0.73, and represented an independent predictor of mortality in this cohort [47].

Urinary NGAL and IL-18 were recently shown to represent early, predictive, sequential AKI biomarkers in children undergoing cardiac surgery [48]. In patients who developed AKI 2–3 days after surgery, urinary NGAL was induced within 2 h and peaked at 6 h, whereas urine IL-18 levels increased around 6 h and peaked at over 25-fold at 12 h postsurgery (AUC 0.75). Both NGAL and IL-18 were independently associated with duration of AKI among cases. Urine NGAL and IL-18 have also emerged as predictive biomarkers for delayed graft function following kidney transplantation [30]. In a prospective multicenter study of children and adults, both NGAL and IL-18 in urine samples collected on the day of transplant predicted delayed graft function and dialysis requirement with AUC of 0.9.

Thus, IL-18 also represents a promising candidate for inclusion in the urinary AKI panel. IL-18 is more specific to ischemic AKI and is not affected by nephrotoxins, chronic kidney disease, or urinary tract infections. It is likely that NGAL, IL-18, and KIM-1 will emerge as sequential urinary biomarkers of AKI.

## Conclusions

The tools of modern science have provided promising novel biomarkers for AKI, with potentially high sensitivity and specificity. These include a plasma panel (NGAL and cystatin C) and a urine panel (NGAL, IL-18, and KIM-1). As they represent sequential biomarkers, it is likely that the AKI panels will be useful for timing the initial insult and assessing the duration of AKI (analogous to the cardiac panel for evaluating chest pain). Based on the differential expression of the biomarkers, it is also likely that the AKI panels will help distinguish between the various types and etiologies of AKI. However, the panels have hitherto been tested only in small studies and in a limited number of clinical situations. It will be important in future studies to validate the sensitivity and specificity of these biomarker panels in clinical samples from large cohorts and from multiple clinical situations. Such studies will be markedly facilitated by the availability of commercial tools for the reliable and reproducible measurement of biomarkers across different laboratories.

**Questions**

(Answers appear following the reference list)

1. Regarding the use of serum creatinine for the diagnosis of acute kidney injury, which of the following statements is true?
  A. Normal serum creatinine increases with age.
  B. Serum creatinine levels are decreased in patients with reduced muscle mass.
  C. In postoperative patients who subsequently develop renal failure, early serum creatinines can be falsely reduced due to vigorous hydration.
  D. In patients with acute renal failure, serum creatinine may not increase for several days after the initial insult.
  E. All of the above.
2. Desirable characteristics of biomarkers of acute kidney injury include all of the following* except*:
  A. They should be noninvasive and utilize easily accessible samples such as blood and urine.
  B. They should be rapidly measurable using a standardized assay method.
  C. They should exhibit high sensitivity and specificity for the detection of acute kidney injury.
  D. They should be measurable in kidney biopsy samples.
  E. They should correlate with clinical outcomes such as mortality, dialysis requirement, length of hospital stay, and morbidity.
3. Regarding traditional urinary biomarkers of acute kidney injury, which of the following statements is true?
  A. Urinary casts are highly sensitive biomarkers of acute kidney injury.
  B. The fractional excretion of sodium provides an accurate measure of acute kidney injury.
  C. Filtered high molecular weight proteins and tubular proteins lack specificity for the early prediction of acute kidney injury.
  D. Hematuria is a highly predictive biomarker of acute kidney injury.
  E. The degree of proteinuria predicts the degree of acute kidney injury.
4. Which of the following constitutes a promising early plasma biomarker of acute kidney injury?
  A. Kidney injury molecule 1 (KIM-1).
  B. Interleukin-18 (IL-18).
  C. Neutrophil gelatinase-associated lipocalin (NGAL).
  D. Serum creatinine.
  E. None of the above.
5. Which of the following constitutes a promising early urinary biomarker panel of acute kidney injury?
  A. Fractional excretion of sodium.
  B. Urine sodium, potassium, creatinine.
  C. Urine NGAL, creatinine.
  D. Urine NGAL, KIM-1, IL-18.
  E. Urine protein, urine blood.

**Answers**

1. E: All of the above
2. D. Kidney biopsies are rarely performed in common clinical situations associated with acute kidney injury because of the invasive nature of this procedure.
3. C: Traditional urinary biomarkers such as albumin and tubular enzymes have not proved to be useful for the early prediction of acute kidney injury.
4. C: NGAL. Cystatin C is also a promising plasma biomarker that rises in tandem following NGAL in several clinical situations that lead to acute kidney injury. KIM-1 and IL-18 are early urinary biomarkers and not plasma biomarkers. Serum creatinine is a late biomarker.
5. D: These represent sequentially expressed biomarkers. Urine NGAL rises rapidly, followed by KIM-1 and IL-18, as has been shown in a variety of clinical situations such as postcardiac surgery, postkidney transplant, and in the intensive care setting. All three biomarkers are robustly detected in the urine several hours to days before the rise in serum creatinine. Emerging preliminary results suggest that urine cystatin C may also be useful in AKI.
